# An Assessment of Participatory Integrated Vector Management for Malaria Control in Kenya

**DOI:** 10.1289/ehp.1408748

**Published:** 2015-04-10

**Authors:** Clifford Maina Mutero, Charles Mbogo, Joseph Mwangangi, Susan Imbahale, Lydia Kibe, Benedict Orindi, Melaku Girma, Annah Njui, Wilber Lwande, Hippolyte Affognon, Charity Gichuki, Wolfgang Richard Mukabana

**Affiliations:** 1 International Centre of Insect Physiology and Ecology, Nairobi, Kenya; 2 University of Pretoria Centre for Sustainable Malaria Control, School of Health Systems and Public Health, University of Pretoria, Pretoria, South Africa; 3 Kenya Medical Research Institute Wellcome Trust Research Programme, Kilifi, Kenya; 4 School of Biological and Life Sciences, Technical University of Kenya, Nairobi, Kenya; 5 School of Public Health, KU Leuven, Leuven, Belgium; 6 The Presbyterian University of East Africa, Nairobi, Kenya; 7 School of Biological Sciences, University of Nairobi, Nairobi, Kenya

## Abstract

**Background:**

The World Health Organization (WHO) recommends integrated vector management (IVM) as a strategy to improve and sustain malaria vector control. However, this approach has not been widely adopted.

**Objectives:**

We comprehensively assessed experiences and findings on IVM in Kenya with a view to sharing lessons that might promote its wider application.

**Methods:**

The assessment used information from a qualitative external evaluation of two malaria IVM projects implemented between 2006 and 2011 and an analysis of their accumulated entomological and malaria case data. The project sites were Malindi and Nyabondo, located in coastal and western Kenya, respectively. The assessment focused on implementation of five key elements of IVM: integration of vector control methods, evidence-based decision making, intersectoral collaboration, advocacy and social mobilization, and capacity building.

**Results:**

IVM was more successfully implemented in Malindi than in Nyabondo owing to greater community participation and multistakeholder engagement. There was a significant decline in the proportion of malaria cases among children admitted to Malindi Hospital, from 23.7% in 2006 to 10.47% in 2011 (*p* < 0.001). However, the projects’ operational research methodology did not allow statistical attribution of the decline in malaria and malaria vectors to specific IVM interventions or other factors.

**Conclusions:**

Sustaining IVM is likely to require strong participation and support from multiple actors, including community-based groups, non-governmental organizations, international and national research institutes, and various government ministries. A cluster-randomized controlled trial would be essential to quantify the effectiveness and impact of specific IVM interventions, alone or in combination.

**Citation:**

Mutero CM, Mbogo C, Mwangangi J, Imbahale S, Kibe L, Orindi B, Girma M, Njui A, Lwande W, Affognon H, Gichuki C, Mukabana WR. 2015. An assessment of participatory integrated vector management for malaria control in Kenya. Environ Health Perspect 123:1145–1151; http://dx.doi.org/10.1289/ehp.1408748

## Introduction

Considerable gains have been made toward achieving the goal of malaria elimination in Africa (WHO 2014; Feachem et al. 2009), but there are growing concerns regarding the sustainability of the vector control interventions whose up-scaling has significantly contributed to this success. In practically every malaria-endemic African country, the primary vector control interventions have been insecticide-based, either using long-lasting insecticidal nets (LLINs) or indoor residual spraying (IRS). Unfortunately, malaria vectors are increasingly becoming resistant to the pyrethroid insecticides that are commonly used with LLINs and IRS (WHO 2012a). According to a recent report, resistance is now widespread and affects all African countries with ongoing malaria transmission (Hemingway 2014).

Furthermore, recent global financial crises have cast serious doubts about the sustainability of current malaria interventions in Africa, given that most funding for malaria control originates from international donors and not from national governments of malaria-endemic countries or other domestic sources (Leach-Kemon et al. 2012; Mutero et al. 2014; Pigott et al. 2012).

Aware of the limitations of exclusive reliance on chemical interventions, the World Health Organization (WHO) has over the years recommended the use of an integrated approach to malaria vector control, involving both chemical and nonchemical methods, including environmental management (Beier et al. 2008; Lindsay et al. 2003; WHO 1982, 2006). The paper “Global Strategic Framework for Integrated Vector Management” (IVM) in 2004 added much-needed clarity to the IVM concept (WHO 2004). It underscored the need for a change in mind-set from the traditional preoccupation with combining the use of only a few limited vector control methods, such as LLINs and IRS, to a comprehensive strategy with key elements that include integration of chemical and nonchemical methods of vector control and their further integration with other aspects of a country’s health-care system, evidence-based decision making, intersectoral collaboration, advocacy and social mobilization, and capacity building. Moreover, IVM can also imply the simultaneous control of multiple diseases transmitted by different vector species in a given area, or one tool controlling several vector-borne diseases transmitted by the same vector (Tusting et al. 2013; WHO 2012b).

Unfortunately, very few practical examples of IVM have been documented. They include IVM implementation at a national level for malaria control in Zambia (Chanda et al. 2008) and at provincial, local-government, and village levels for control of malaria and dengue in the Philippines (van den Berg et al. 2012). The purpose of this article is to disseminate in detail the findings of a comprehensive assessment of community-level IVM for malaria control in Kenya. The assessment is intended to share experiences and lessons toward further development, promotion, and adoption of IVM.

## Methods

Our assessment was based on information derived from two main sources: *a*) a qualitative external evaluation in May 2012 of IVM implementation at two different geographic locations in Kenya between 2006 and 2011 (ICIPE 2012), and *b*) an analysis of entomological and malaria case data collected by the two projects. During the external evaluation, the following were used as the IVM process or outcome indicators (WHO 2004, 2012c): evidence-based decision making, integrated approaches, advocacy and social mobilization, intra- and intersectoral collaboration, and capacity building.

*Study sites*. The study sites were Malindi in coastal Kenya (3°13´ S; 40°7´ E) and Nyabondo in the western part of the country (0°22´ S; 34°58´ E). Both sites are within Kenya’s endemic malaria zone with all-year risk of malaria transmission (DOMC 2009). Malindi is a major tourist destination and receives thousands of tourists from different parts of the world every year. The IVM project there sampled mosquitoes from urban, peri-urban, and rural settings and collated malaria data from the main Malindi subcounty hospital. The classification into urban, peri-urban, and rural was based on a sampling strategy previously applied in Malindi (Keating et al. 2003). The main difference among the three strata was in their level of development and system of physical planning. Thus, the urban area was characterized by paved roads, piped water, planned housing, drainage services, and electricity lines, whereas the rural area was mainly farmland with houses without electricity or piped water. The peri-urban area was transitional between the urban and rural settings and had pockets of farming and a patchy distribution of piped water and electricity. The whole area under study in Malindi was approximately 32 km^2^. For purposes of mosquito sampling, it was divided into 1 km × 1 km grids, hereafter referred to as “cells.” Nyabondo is, on the other hand, a rural plateau area in Kisumu County, about 30 km northeast of Lake Victoria. Brickmaking is the main economic activity. The project covered a 30-km^2^ area, which had been divided into cells similar to those in Malindi. Adult mosquitoes were sampled from houses within cells located in the following four locations of Nyabondo: South Nyakach, Oboch, South West, and Sigoti. Malaria data were from three local health facilities: Nyabondo Mission Hospital, Nyakach AIC Dispensary, and Sigoti Health Centre.

*Qualitative external evaluation*. The evaluation assessed the IVM implementation process including its impact on various direct beneficiaries, among them, communities, community-based organizations (CBOs), local authorities, schools, and research institutes. External evaluation methods consisted of institutional analysis involving website review and any other information on the institutions that supported IVM operational research technically and financially at the two project sites. It also included documentary analysis of project proposals and progress reports in addition to interviews with project staff to draw out their experiences in the field and investigate whether the projects would be self-sustaining should the current external operational research-based funding be discontinued. Interviews with community groups also formed part of the external evaluation, primarily as participatory evaluation discussions but also as individual interviews with group leaders and recipients of the IVM technology. A field visit to the Malindi IVM project site was conducted for direct observation of interventions and interviews with the project staff, community group leaders, and community members as recipients of the IVM approach to establish how the community related with the research staff, what new knowledge they acquired and whether they were making use of it, as well as what other benefits accrued from the project’s intervention. Last, a brainstorming retreat with research teams for the two projects was held to draw out the lessons learned from the projects since their inception.

*Analysis of entomological and malaria case data*. Data background. The projects had conducted entomological sampling on a monthly basis each year. CDC light traps (LTs) and pyrethrum spray catches (PSCs) (Silver 2008) had been used to collect adult mosquitoes from inside houses in Malindi, whereas only LTs had been used in Nyabondo. Up to three LTs had been deployed per cell in both Malindi and Nyabondo, sometimes twice in a month. In the case of Malindi, the PSC sampling undertaken during 2010 and 2011 had involved day-time collection of mosquitoes from up to 10 houses from each cell once a month.

Data on malaria cases had been collated by the projects from laboratory and outpatient registers, at the local government hospital in Malindi and at two government health facilities (Nyakach AIC Dispensary and Sigoti Health Centre), and at one privately owned hospital (Nyabondo Hospital) in Nyabondo. Malaria diagnosis had been by microscopy in all the facilities. The health facilities used for data collation were similar in Malindi and Nyabondo, being either run by the Ministry of Health (MoH) or faith-based. In Kenya, malaria treatment for children under 5 years of age is free of charge, including diagnostics and medicine (DOMC 2009). The medical record storage with the MoH facilities is standardized. Health seeking in Malindi and Nyabondo areas is primarily from MoH facilities because they are either free or charge minimal user fees on some of their services. The malaria case data for Nyabondo were from outpatients of all ages, whereas for Malindi, the case data were only from children 0–14 years of age who were admitted to Malindi district hospital. Incompleteness of outpatient malaria data in Malindi and inpatient data in Nyabondo led to these sets of data being left out of the analyses.

Statistical analysis. Densities of *Anopheles* and culicine mosquitoes and the number of malaria cases were analyzed to serve as the entomological and epidemiological indicators of change during the projects’ implementation period. The data for Malindi were for the period 2006–2011, but equivalent data for Nyabondo were only available for the period 2009–2011.

Statistical analyses were performed using STATA (version 10.1). In Malindi, the project used different techniques to sample adult mosquitoes from 2006 to 2009 (LT) and 2010 to 2011 (PSC); therefore, the corresponding data sets were analyzed separately. Proportions were compared using chi-square test. To analyze relative density of adult mosquitoes over the period while controlling for area and/or month, a zero-inflated negative binomial (ZINB) model was fitted. This was done separately for anophelines and culicines. The variable area had three categories in Malindi: urban, peri-urban, or rural. Risk ratios (RR) were computed for each year in comparison to 2006 and to urban in the case of area. The Kruskal–Wallis test was used in situations that violated model distributional assumptions and had model convergence problems due to small numbers of mosquito counts. In Nyabondo, a zero-inflated Poisson (ZIP) model was fitted to study the association between adult mosquito abundance and year while adjusting for site and month, with “cell” (i.e., 1 km × 1 km grid) as the cluster. For both Malindi and Nyabondo, only female mosquitoes were included in the analyses because they are responsible for disease transmission. Zero-inflated count models were fitted to account for overdispersion (meaning that the variability encountered in the data is not equal to the mean, as prescribed by the Poisson distribution) and excess zeroes. Count data are most commonly modeled using the Poisson model or negative binomial model. In substantive research, some data, however, come with a high percentage of zero counts—far more than are accounted for by the Poisson or negative binomial distributions. When this occurs, ZIP and ZINB models, which are extensions of the Poisson and negative binomial models, respectively, are often used to account for the excess zeroes. For brevity, these models assume that data come from a mixture of two distributions in which the structural zeroes from a binary distribution are mixed with the non-negative integer outcomes from a count distribution. The structural zeroes are typically modeled using logistic (or probit) regression, and the count outcomes are modeled using Poisson or negative binomial regression. Thus, a zero-inflated (ZIP or ZINB) model has both the count (non-zero) part and a binary (zero) part, with the latter describing the change in odds for always having zero counts (Hilbe 2011). For Malindi, a ZINB model was preferred to ZIP model (likelihood-ratio test *p* < 0.0001).

## Results

*IVM site 1: Malindi*. Adult mosquito abundance and malaria cases. Of 83,146 female mosquitoes collected in Malindi between 2006 and 2011, only about 1% were malaria vectors (274 *Anopheles gambiae* and 6 *Anopheles funestus*), whereas 98.9% (82,260) comprised culicines, mainly *Culex quinquefasciatus*. The total trap-nights in 2006, 2007, 2008, and 2009 were 990, 1,536, 1,437, and 623, respectively. For PSC, the number of house-days in 2010 and 2011 were 219 and 540, respectively. Mosquito abundance generally declined over the years, with culicines remaining dominant throughout the study period (Table 1). *An. gambiae* were uniformly very low over a period of 3 years from September 2008 to September 2011, coinciding with up-scaling of vector control interventions and mobilization of communities in Malindi through a partnership, PUMMA [Punguza Mbu na Malaria Malindi (Eliminate Mosquitoes and Malaria from Malindi)], which included several community-based groups in Malindi, the Kenya Medical Research Institute (KEMRI), International Centre of Insect Physiology and Ecology (ICIPE), Biovision Foundation, and Kenya’s MoH, among others (ICIPE 2012). During the same period, the proportion of malaria cases among children admitted at Malindi Hospital declined significantly from 23.7% in 2006 to 10.47% in 2011 (*p* < 0.001) (Table 1).

**Table 1:** Malindi data: adult mosquito relative density (total collected) and percentage of malaria cases (total admissions) among children 0–14 years of age.

Sampling method/year	*An. gambiae*	*An. funestus*	Culicines	Other species	Percent malaria cases in children (no. of admissions)
CDC LT^*a*^
2006	0.196 (194)	0.006 (6)	24.47 (24,230)	0.24 (241)	23.65 (2,436)
2007	0.033 (51)	0 (0)	15.51 (23,784)	0.16 (240)	20.06 (2,408)
2008	0.018 (26)	0 (0)	18.40 (26,438)	0.05 (71)	16.64 (2,151)
2009	0 (0)	0 (0)	10.66 (6,639)	0 (0)	15.59 (2,732)
PSC^*b*^
2010	0 (0)	0 (0)	1.25 (274)	0.36 (79)	13.05 (2,367)
2011	0.006 (3)	0 (0)	1.66 (895)	0.004 (2)	10.47 (2,283)
Abbreviations: CDC LT, CDC light trap; PSC, pyrethrum spray catches. ^***a***^CDC LT sampling (2006–2009), data represent the average number of mosquitoes/trap/night (total number collected at all locations per year). ^***b***^PSC sampling (2010–2011), data represent average number of mosquitoes/house/day (total number of mosquitoes from all locations).					

The Kruskal–Wallis test results indicated a significant decline in the density of *An. gambiae* over the years (*p* = 0.001). For culicines, the ZINB model results indicated a significant association between vector abundance and year. Compared with 2006 and after controlling for month, there was a significant reduction in mosquito abundance in 2007, 2008, and 2009 [for 2007, RR = 0.60, 95% confidence interval (CI): 0.53, 0.68; 2008, RR = 0.72, 95% CI: 0.64, 0.82; and 2009, RR = 0.56, 95% CI: 0.46, 0.68]. The binary equation section, which describes the change in odds for always having zero mosquito counts versus not having zero counts, indicated that all the years had greater odds of zero mosquito counts than 2006, which further supports the observations made above that the mosquito counts decreased over the years.

Evidence-based decision making and integrated approaches. The overall malaria IVM strategy and interventions in Malindi were guided by operational research evidence. Specific vector control interventions included MoH-led distribution of LLINs, community-driven mosquito larval source management (LSM) through environmental management and the application of biolarvicides [*Bacillus thuringiensis israelensis* (Bti)] (Fillinger et al 2003; Kibe et al. 2006; Mwangangi et al. 2011), and community education through neighborhood campaigns and school-based school health clubs with the motto “children as agents of change in malaria and mosquito control.” Implementation of the various interventions was spearheaded by mosquito scouts, each assigned a “cell” (i.e., a 1 × 1 km grid) to survey adult and larval mosquito densities, mobilize and educate the community about IVM, and organize neighborhood campaigns and school-based health and environmental clubs. About 16 scouts participated each year, the majority of whom were adult women. The scouts were mainly drawn from among community-based health workers and already existing antimosquito and malaria organizations, most notably, PUMMA. Community members participated in the actual elimination of the majority of breeding sites through filling up or draining stagnant pools of water, getting rid of waste plastic containers, and covering water wells, toilets, and household water storage containers.

Advocacy and social mobilization. The Malindi project was made visible through awareness creation on designated days, which received media (radio and television) coverage during a particular year. On such occasions the project held exhibitions and demonstrations and participated in radio and television documentaries. The project also had school clubs compete, with trophies given for exemplary malaria control activities, including singing songs, participating in drama, citing poetry, and developing articles about mosquitoes. Schools were also involved in making objects using waste plastic collected from the environment as a means of destroying potential mosquito breeding sites. The media’s attention on community mosquito and malaria control in Malindi raised the town’s profile and in turn led to the Municipal Council actively supporting the activities including, in certain cases, overseeing the covering and elimination of *Anopheles* breeding sites.

The mosquito scouts—having been trained in the identification of mosquitoes, their breeding sites, and their elimination—gained community interactive skills, community trust, and respect, thereby raising their self esteem and social capital. Thus, because of this training, about 15 of a total of 62 scouts ended up finding gainful employment in other projects and organizations. Ultimately this served as a motivation for other people to be involved in mosquito control activities. The groups directly involved in mosquito control, such as PUMMA, developed income-generating activities around mosquito control activities, including using waste plastic paper to make baskets, poles, and blocks, which they would later sell. This connection of mosquito control to income generation uplifted the socioeconomic status of the people involved and became a central activity for a number of youth groups in Malindi. The fact that a portion of the income generated by these groups was dedicated to mosquito and malaria control augured well for local ownership and sustainability of the malaria control efforts.

Interviews with community groups during the qualitative external evaluation established that the communities could clearly see the benefits of income generation. Community members testified that they would not stop the mosquito control and associated income- generating activities, even in the event of the external project funding coming to an end. However, the presence of the project institutions KEMRI and ICIPE was viewed as being essential, particularly for liaison with the Municipal Council, MoH, and ministries of fisheries, environment, and natural resources. In the words of several mosquito scouts, the project institutions were like parents, whose authoritative input was taken seriously by all stakeholders. Thus, in spite of the Malindi project having formed a stakeholders’ forum that could take over this role, it was clear from interviews with the various stakeholders that continued presence of the research institutions was needed as a means of consolidating the authority and ownership of the activities by the stakeholders and the recipients.

Intersectoral collaboration. The institutional partnership forged by the Malindi project clearly demonstrated that malaria was not an exclusive priority of the health sector, but rather an intersectoral issue involving a range of actors that could be conveniently grouped into various categories based on their respective complementary roles.

Capacity building. Capacity building at the grassroots community level centered around the mosquito scouts, who in turn became trainers for the various community groups, school clubs, and households, and generally mobilized neighborhood campaigns for elimination of mosquito breeding sites. It is also through the mosquito scouts that continuous training on proper usage of LLINs was carried out in urban and peri-urban areas of Malindi. Furthermore, the ICIPE and KEMRI trained two PhD and five MSc students as well as four interns (postgraduate diploma in health research methodology) within the project.

*IVM Site 2: Nyabondo*. Adult mosquito abundance and malaria cases. The total mosquito trap-nights in 2009, 2010, and 2011 were 1,020, 1,040, and 1,018, respectively. Of 42,435 female adult mosquitoes collected, 10.4% were *An. gambiae sensu lato,* 0.2% *An. funestus*, and 88.5% culicines—mainly *Cx. quinquefasciatus*. The remaining 0.9% consisted of other species, which included *Anopheles coustani* and *Anopheles pharaoensis*. A subsample of *An. gambiae s.l.* was further analyzed for species identification, and the results indicated that 99.3% were *Anopheles arabiensis*, while 0.7% were *An. gambiae sensu stricto*. The relative density of adult mosquitoes and malaria case data for each year are shown in Table 2. More *An. gambiae* were collected in 2010 compared with 2009 and 2011 in all the study villages. In contrast, culicine density showed an increasing trend from 2009 to 2011. Table 2 also summarizes the number of patients and malaria cases observed at each health facility in Nyabondo by year. Data for Sigoti Health Centre were not available during 2010. Of 10,348 patients attending the three facilities over a 3-year period, 29.5% (95% CI: 28.6, 30.4) were malaria cases. This proportion was 24.5%, 34.7%, and 30.3% in 2009, 2010, and 2011, respectively.

**Table 2:** Nyabondo data: average number of adult mosquitoes/trap/night (total number collected per year) by site collected using CDC light traps, and percentage of malaria cases among all outpatients (total number of all outpatients) by hospital.*^a^*

Year	*An. gambiae*				Culicines				Percent malaria cases (no. of outpatients)			
	South Nyakach	Oboch	South West	Sigoti	South Nyakach	Oboch	South West	Sigoti	Nyabondo	Nyakach AIC	Sigoti	Combined
2009	0.56 (171)	0.64 (131)	0.17 (43)	0.20 (50)	3.77 (1,155)	10.84 (2,211)	5.84 (1,488)	7.50 (1,912)	14.32 (1,822)	61.13 (391)	27.26 (1,570)	24.53 (3,783)
2010	2.18 (681)	3.48 (723)	2.92 (758)	2.95 (768)	6.24 (1,953)	11.98 (2,492)	9.90 (2,573)	19.10 (4,966)	13.74 (1,739)	61.80 (1,343)	NA	34.69 (3,082)
2011	0.30 (91)	2.04 (417)	0.28 (66)	1.85 (513)	12.61 (3,834)	16.63 (3,393)	14.75 (3,422)	29.30 (8,146)	14.32 (1,767)	63.30 (902)	28.26 (814)	30.26 (3,483)
Abbreviations: NA, not available. No malaria data were available for Oboch. ^***a***^The malaria cases data were from all outpatients of all ages.												

Table 3 summarizes the ZIP model results for adult *An. gambiae* and *Culex* spp. For *An. gambiae* there was a significant association between vector abundance and year. Compared with 2009 and after adjusting for site and month, there were significantly more mosquitoes in 2010. There were also more mosquitoes in 2011 than in 2009, but this difference was not significant. On the other hand, of the four study sites in Nyabondo, South Nyakach had the lowest mosquito density, although the difference was only significant for Oboch. For *Culex* spp., there was also a significant association between vector abundance and time—with both 2010 and 2011 recording significantly more mosquitoes than 2009, after adjusting for site and month of the year. As was the case with *An. gambiae*, all the other three sites recorded more *Culex* spp. than South Nyakach. The binary model part for both *An. gambiae* and *Culex* spp. indicated that year was the only significant predictor of excess zeroes—although the direction of the effect was opposite to the project’s expectation; that is, there was an increase in the number of mosquitoes over time.

**Table 3:** Nyabondo adult mosquito data: adjusted RRs and odds ratios (ORs) (95% CIs) from ZIP model for *An. gambiae* and culicines.*^a^*

Variable	*An. gambiae*		Culicines	
	RR or OR (95% CI)	*p*-Value	RR or OR (95% CI)	*p*-Value
Poisson part
Year
2009	1.00		1.00
2010	3.07 (2.08, 4.53)	< 0.001	1.65 (1.2, 2.26)	0.002
2011	1.54 (0.72, 3.29)	0.263	2.32 (1.57, 3.43)	< 0.001
Site
South Nyakach	1.00		1.00
Oboch	1.66 (1.09, 2.53)	0.018	1.78 (1.13, 2.81)	0.014
South West	1.35 (0.64, 2.83)	0.431	1.36 (0.93, 1.98)	0.116
Sigoti	1.82 (0.8, 4.15)	0.156	2.39 (1.53, 3.73)	< 0.001
Month
January	1.00		1.00
February	0.20 (0.12, 0.34)	< 0.001	0.52 (0.34, 0.79)	0.002
March	0.31 (0.22, 0.45)	< 0.001	0.80 (0.55, 1.17)	0.247
April	0.95 (0.49, 1.85)	0.873	1.68 (1.17, 2.42)	0.005
May	2.32 (1.37, 3.92)	0.002	2.00 (1.57, 2.55)	< 0.001
June	1.75 (1.12, 2.73)	0.014	2.31 (1.47, 3.64)	< 0.001
July	0.82 (0.50, 1.34)	0.425	1.41 (0.91, 2.17)	0.124
August	0.44 (0.22, 0.88)	0.020	0.72 (0.43, 1.19)	0.196
September	0.90 (0.58, 1.41)	0.655	1.43 (1.03, 1.98)	0.034
October	1.25 (0.68, 2.30)	0.463	0.69 (0.48, 1.00)	0.052
November	1.34 (0.76, 2.34)	0.311	0.70 (0.45, 1.08)	0.104
December	1.06 (0.65, 1.73)	0.814	0.81 (0.62, 1.06)	0.121
Binary part
Year
2009	1.00		1.00
2010	0.32 (0.25, 0.41)	< 0.001	0.78 (0.62, 0.97)	0.029
2011	0.42 (0.28, 0.64)	< 0.001	0.45 (0.34, 0.59)	< 0.001
Site
South Nyakach	1.00		1.00
Oboch	0.74 (0.52, 1.04)	0.083	1.09 (0.87, 1.35)	0.463
South West	1.56 (1.02, 2.40)	0.042	1.11 (0.89, 1.37)	0.355
Sigoti	1.29 (0.78, 2.13)	0.322	0.81 (0.49, 1.33)	0.403
^***a***^Both Poisson model and binary model are parts of the same ZIP model. The Poisson part models the non-negative count catches, and the binary part models the structural zeroes. The estimates from the Poisson part are RRs, and estimates from binary part are ORs. The Poisson part had three covariates (i.e., year, site, and month), and the binary model part had two covariates (i.e., year and site). Reference levels were 2009, South Nyakach, and January for year, site, and month, respectively.				

*Evidence-based decision making*. The qualitative evaluation found that a baseline survey previously carried out by the project had revealed that 74% of the respondents perceived malaria as a health risk associated with brickmaking. The survey had been conducted in early 2012, in which 186 brickmakers were interviewed to determine whether they associated brickmaking with malaria transmission.

The survey further found that *Anopheles* mosquito larvae were common in flooded brick pits and abandoned fish ponds, indicating that these man-made habitats posed a risk of malaria transmission. In addition, the respondents believed that IVM involving planting of trees (35%), draining of abandoned ponds (24%), and back-filling of brick pits with broken bricks (20%) would be among the best ways to tackle mosquito breeding. The decision to use fish predators against mosquito larvae was being explored based on the observation that ponds that had been stocked with fish were larvae free. These findings indicate that the decisions to start the malaria IVM in Nyabondo was evidence based.

Integrated approaches. Like in Malindi, mosquito breeding sites in Nyabondo were treated with biolarvicides (Bti and/or neem extracts), manipulated to create or improve drainage (water management), or filled up to eliminate them. Fish were introduced into ponds to serve as predators of mosquito larvae. The community was mobilized to participate in these activities as well as those related to use of LLINs and IRS.

Advocacy and social mobilization. In spite of many challenges, including an expectation by the community to be paid for participating in project activities, the Nyabondo project managed to recruit and train mosquito scouts and reach and involve school health clubs and health club patrons in mosquito and malaria control. The project worked with government ministries toward initiating a reclamation of abandoned fish ponds and also involved two schools in fish farming. Moreover, the project conducted community training and developed and distributed information, education, and communication materials annually and also broadcast mosquito and malaria control messages via radio.

Intersectoral collaboration. The project connected with various ministries: the Ministry of Fisheries for fish farming, the Ministry of Education for school health clubs and patrons (school health clubs were made up of pupils, with the teacher in-charge acting as the patron), the MoH to distribute LLINs and monitor malaria prevalence, and the Ministry of Agriculture for land reclamation. It also formed an umbrella community-based organization (CBO) dealing with mosquito control, from which it drew mosquito scouts. Activities of this CBO were integrated with those of the health club’s patrons committee, thereby giving the project invaluable gains. However, at the time of the qualitative external evaluation in May 2012, more efforts were needed to consolidate the gains because many of the community participants in Nyabondo still felt that they needed to be financially compensated for participating in project activities. They still felt as if they were providing a service to an external entity without clearly seeing its benefits.

*Capacity building*. Several community training sessions were carried out annually, which built capacity for mosquito control, resource mobilization, and fish farming. In addition, annual exchange visits were made to the Malindi IVM project. At least one mosquito scout, two other community members, and one health club patron went on the exchange visit in a particular year, with the chance being given to another similar group the following year. Through the school health clubs and patrons, 14 schools had, by the time of the qualitative external evaluation, been trained on mosquito and malaria control and 3,000 members of the community trained to recognize and eliminate man-made mosquito breeding sites.

## Discussion

The present assessment compares the relative success of implementing IVM for malaria control at two project sites in Kenya. The projects evolved out of the need to translate scientific knowledge on proven and well-known malaria vector control interventions into practical solutions at the community level. Failure to implement known solutions has previously been identified as a major bottleneck to improvement of human health in spite of substantial research, especially in Africa (Sanders and Haines 2006). The purpose of the research was to provide technical support and guide the implementation of a range of activities, including income generation, and not necessarily to compare the effectiveness of specific vector control interventions such as LLINs and larviciding. Consequently, the holistic community-development approach of the projects allowed only the use of qualitative and descriptive analysis of the social, institutional, and management aspects of IVM and limited quantitative evaluation of entomological and malaria case data. A more rigorous statistical analysis of the effectiveness and impact of a range of specific IVM interventions would have been possible if the projects had been designed as cluster-randomized controlled trials (Kramer et al. 2014). However, this option had not been explored by the respective researchers at the onset of the projects largely due to constraints in research funding.

Given the qualitative nature of the research, the significant decline in malaria and malaria vectors that the comprehensive assessment found to be associated with IVM interventions in Malindi needs to be interpreted with caution, particularly because the project took place at a time when there was also routine MoH-led mass distribution of LLINs in Kenya (Noor et al. 2007). The discussion of the quantitative results is, therefore, deliberately limited to noting the significant decline of malaria and malaria mosquitoes in Malindi and the lack of any similar changes in Nyabondo.

Based on the qualitative analysis, IVM operations were more successful and sustainable in Malindi than in Nyabondo. The obvious explanation for this difference was the existence in Malindi of a well-organized system of multistakeholder collaboration, advocacy, and social mobilization. For instance, this situation seemingly boosted the use of LLINs and environmental management in Malindi throughout the project period. From the assessment, it was evident that the respective roles of government departments, national and international research institutes, nongovernmental organizations (NGOs), and communities were all important factors for successful malaria IVM. A replication of the Malindi IVM model in other parts of Kenya would most likely require an effective national policy to promote and support the implementation of a multisectoral approach to malaria (RBM 2013).

The results for Malindi were, to a large extent, in agreement with those from the Philippines, where involvement of local authorities and empowerment of communities were among the key factors found to contribute to improved efficiency and sustainability of vector control operations (van den Berg et al. 2012). Elsewhere, in Uganda, respondents in a survey to assess perceptions of various stakeholders indicated that community participation in IVM would be at its strongest when the government was also involved (Mutero et al. 2012). The greatest need for continued engagement of government and international and national research agencies is perhaps in connection with the long-term surveillance, monitoring, and evaluation that are required, using standard indicators for malaria IVM (WHO 2012c).

A further explanation of the greater success achieved in mobilizing communities and other stakeholders in Malindi may lie in the fact that it is an urban and peri-urban area and is therefore easier to target with advocacy and other information than Nyabondo, which is largely rural. Moreover, the Malindi project was also better resourced than the one in Nyabondo, as the former had supplementary funding from other donors besides Biovision Foundation. More research is needed to clearly identify and address behavioral and other barriers hindering optimal participation by communities, particularly in the more rural and resource-scarce settings. For instance, in Nyabondo, most of the brickmakers were not members of that community but had rented plots of land from which they made their livelihoods. Follow-up research would be needed to better understand how the brickmakers’ attitudes and behavior may have affected the IVM results.

Besides the vertical collaboration observed among stakeholders from the community to policy-making levels, horizontal collaboration and integration were discernible within each of the three levels. At the policy level, the MoH in Malindi closely worked with the Ministry of Local Government, represented by the Malindi Municipal Council. Furthermore, the Ministry of Tourism was also either directly or indirectly involved through the hotel industry. Collaboration by the three ministries suited the urban and tourism setting of Malindi and could likely be replicable in other similar settings along the eastern African coast. Potentially, further awareness would be needed among the hotel industry actors in Malindi to empower them to contribute more effectively to malaria IVM, for instance, through proper management of swimming pools so that they do not turn into mosquito breeding sites during the off-season for tourists (Impoinvil et al. 2008). Tourism was also found to contribute to IVM through supporting local enterprises with potential for generating income for sustaining mosquito control. This was evident from interviews with hoteliers during the qualitative external evaluation and the research itself.

In Nyabondo, collaboration was mainly between the MoH and the Ministry of Fisheries because of the potential role of fish farming in aggravating malaria risks in the area. Nyabondo clearly illustrated the case where policies and activities of a non–health sector (i.e., fisheries) have important implications for malaria vector control. Engaging the Ministry of Fisheries was essential in Nyabondo in order to forestall a proliferation of poorly maintained or disused fish ponds, as they have been shown to significantly contribute to an increase in malaria vector populations in the study area (Howard and Omlin 2008; Imbahale et al. 2013). A comparable situation has been reported in the Mekong Delta area of Southeast Asia, where poorly managed ponds for shrimp cultivation have been associated with malaria risks (Lindsay et al. 2004). However, although the use of larvivorous fish for mosquito control has been practiced for many years in different parts of the world (WHO 2003), the actual impact of fish on malaria transmission is yet to be established (Walshe et al. 2013). The examples of Malindi and Nyabondo, in a sense, highlighted the need for prospectively assessing the health impact of development projects, including those respectively dealing with tourism and fish farming (Birley 2011).

Regarding horizontal collaboration at the research level, the close partnership forged by an international research organization (ICIPE), a national research institute (KEMRI), and an international research and development NGO (Biovision Foundation) constituted an important conduit and intermediary for two-way linkages and communication between the community and policy-making levels. The role of the research institutes was highly regarded in Malindi and was considered as part and parcel of malaria IVM. The institutional partnership, among other things, facilitated a transdisciplinary approach to malaria by combining ICIPE’s expertise in integrated vector and pest management with the biomedical expertise of KEMRI and the social science and development approaches of Biovision Foundation. Transdisciplinary approaches have previously been suggested as being crucial for sustainable malaria control in Kenya (Mutero et al. 2004).

At the community level, the two projects demonstrated a hitherto unexplored opportunity for horizontal collaboration in the form of recruitment of mosquito scouts to actively network and virtually sensitize all segments of the community and community-based organizations regarding malaria control. This arrangement was highly successful in building the relevant capacity in Malindi. It also proved easy to replicate in Nyabondo, albeit on a smaller scale. Chief among the groups participating actively at the community level were school clubs through extracurricular activities related to health and environment. Creating local networks involving mosquito scouts and school clubs proved to be an effective way of promoting IVM, a practice that would be worth initiating and evaluating in other settings. In the case of Malindi, the very low levels of malaria vectors and malaria prevalence observed during the intervention period became a major incentive for communities and other stakeholders to continue engaging in IVM. However, important lessons can be learned from previous work in Dar es Salaam, Tanzania, regarding other options of sustaining the motivation and participation of community-recruited IVM workers (Chaki et al. 2011). According to the Dar es Salaam study, recruiting a few well-remunerated community workers in that particular setting was more practical and, therefore, a better strategy than relying entirely on a host of unpaid and unemployed volunteers. The Tanzanian experience might be especially relevant for Nyabondo, where the community was found to expect monetary compensation for participating in project activities. It is worth noting here that there was also no direct financial compensation of communities in Malindi, yet the response to IVM was more positive compared with Nyabondo.

Finally, it was evident in both Malindi and Nyabondo that in spite of the IVM operations and existing wide coverage with LLINs, *Cx. quinquefasciatus—*a mosquito species that does not transmit malaria—persisted in relatively much higher numbers than those of the malaria vector, *An. gambiae*. Paradoxically, the presence of the ubiquitous *Cx. quinquefasciatus* and its attendant biting nuisance might be beneficial in encouraging people to continue using LLINs for malaria control in areas where malaria vector populations are extremely low due to interventions. In the absence of the nuisance of mosquitoes biting, people tend to cease using bed nets because of an erroneous assumption that very low anopheline populations would not pose a significant risk of malaria (Pulford et al. 2011).

## Conclusions

Sustainability of IVM for malaria control at a community level was found to be dependent on active participation by community-based groups and their collaboration with NGOs, international and national research institutes, and various government ministries. The results support a previous view that developing dynamic and integrated health innovation systems involving scientific and policy institutions, as well as other stakeholders, is essential for creating sustainable health care systems in Africa (Chataway et al. 2009).
